# The Cell Surface Proteome of Human Mesenchymal Stromal Cells

**DOI:** 10.1371/journal.pone.0020399

**Published:** 2011-05-26

**Authors:** Christian Niehage, Charlotte Steenblock, Theresia Pursche, Martin Bornhäuser, Denis Corbeil, Bernard Hoflack

**Affiliations:** 1 Biotechnology Center, Dresden University of Technology, Dresden, Germany; 2 Department of Hematology and Oncology, University Hospital Dresden, Dresden, Germany; University of South Florida, United States of America

## Abstract

**Background:**

Multipotent human mesenchymal stromal cells (hMSCs) are considered as promising biological tools for regenerative medicine. Their antibody-based isolation relies on the identification of reliable cell surface markers.

**Methodology/Principal Findings:**

To obtain a comprehensive view of the cell surface proteome of bone marrow-derived hMSCs, we have developed an analytical pipeline relying on cell surface biotinylation of intact cells using cell impermeable, cleavable sulfo-NHS-SS-biotin to enrich the plasma membrane proteins and mass spectrometry for identification with extremely high confidence. Among the 888 proteins identified, we found ≈200 *bona fide* plasma membrane proteins including 33 cell adhesion molecules and 26 signaling receptors. In total 41 CD markers including 5 novel ones (CD97, CD112, CD239, CD276, and CD316) were identified. The CD markers are distributed homogenously within plastic-adherent hMSC populations and their expression is modulated during the process of adipogenesis or osteogenesis. Moreover, our *in silico* analysis revealed a significant difference between the cell surface proteome of hMSCs and that of human embryonic stem cells reported previously.

**Conclusions/Significance:**

Collectively, our analytical methods not only provide a basis for further studies of mechanisms maintaining the multipotency of hMSCs within their niches and triggering their differentiation after signaling, but also a toolbox for a refined antibody-based identification of hMSC populations from different tissues and their isolation for therapeutic intervention.

## Introduction

Multipotent human mesenchymal stromal cells (hMSCs) [1], initially described as colony-forming unit-fibroblasts [2], [3], are non-hematopoietic progenitors found in many tissues such as bone marrow, umbilical cord blood, adipose tissues [4], dermis, muscles [5], and placenta [6]. They are self-renewing cells that can differentiate into a variety of cell types including osteoblasts, chondrocytes, and adipocytes [7], [8] and possibly neuron-like cells [9], [10], hepatocytes [11], or pancreatic-like cells [12]. Due to their multi-lineage differentiation potential and their ability to migrate to injured tissues [13], hMSCs are considered as promising candidates for tissue engineering and regenerative medicine. Their properties to suppress responses linked with immunity [14] or inflammation [15], [16], [17] is also an advantage for clinical applications. Beside ethical issues, pluripotent embryonic stem cells (ESCs) have also these abilities and could also be considered for therapeutic intervention. However, donor-derived tumors have been observed after ESC transplantation [18].

Bone marrow-derived hMSCs have been isolated based on their ability to adhere onto plastic surfaces [19]. These plastic adherent cells can easily be expanded *ex vivo* while maintaining their undifferentiated phenotype and gene expression profile during long-term expansion [20]. However, some particular markers (e.g. CD133 (prominin-1)) can be lost [21], possibly due to methods used for either their isolation or propagation in culture [22], [23], [24]. Until now, no reliable cell surface markers have been described in freshly isolated hMSCs.

It has always been difficult to identify membrane proteins by mass spectrometry (MS), in particular plasma membrane proteins that can be used as cell surface markers [25], [26]. Different methods have been applied to enrich membrane proteins, in particular cellular fractionation [27], [28]. However, the complexity of the resulting proteome including quantitative proteomic analysis of hMSCs undergoing differentiation towards distinct cell lineages [27], [28] is usually underestimated due to abundant contaminants, and therefore a comprehensive understanding of the cell surface proteome is limited [29].

Here, we have combined cell biological, biochemical and analytical methods allowing us to present the most comprehensive cell surface proteome of hMSCs available to date. This data set can be used to solve basic questions concerning the molecular and cellular biology of hMSCs and their applications in regenerative medicine.

## Results

### Protein isolation and identification

**Figure 1:**
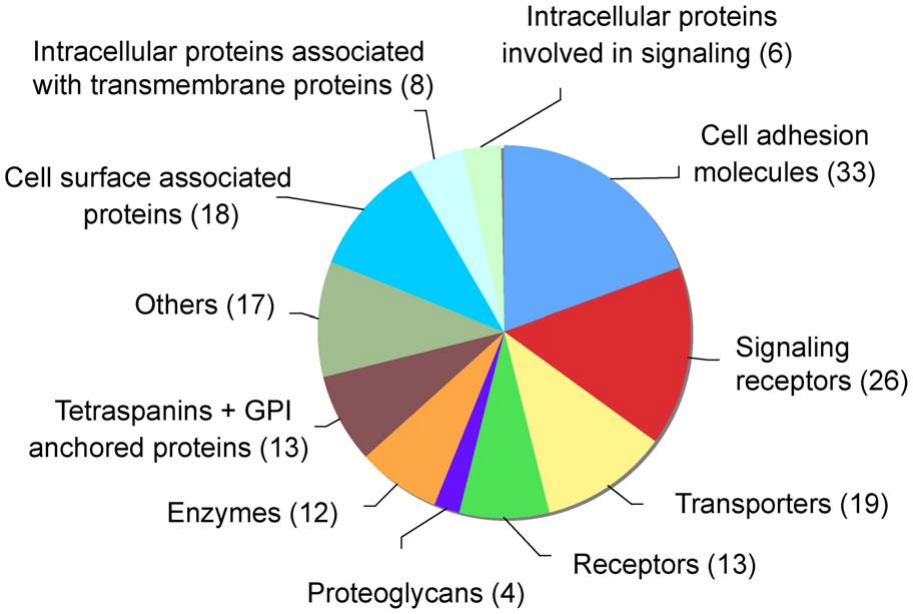
Cell surface proteome of human mesenchymal stromal cells. Distribution and functional clustering of identified proteins.

Several methods have been used to reveal a cell surface signature of hMSCs; first, cellular fractionation (purification of microsomal membranes) followed or not by 1D-gel and MS-based identification [27], [28] and second, 2D-gel analysis using intact cells and MS-based identification [30], [31], [32]. To identify *bona fide* cell surface proteins, we have chosen a different approach relying on the biotinylation of intact cells using cell impermeable, cleavable sulfo-NHS-SS-biotin. After harvesting of the cells, the biotinylated proteins were purified on streptavidin-beads. Upon reduction, the biotinylated proteins were released from the beads and their adsorbed contaminants. The eluted material was further fractionated by 1D-PAGE and, after in gel-trypsinization, the 24 slices of the lanes were analyzed by LC-MS/MS. The data from the LC-MS/MS analysis gave rise to a unique data set of 888 proteins. The data set represents proteins that were identified with at least two sequenced peptides detected with high mass accuracy. Functional classification of the proteins according to the Gene Ontology database and literature surveys revealed 169 *bona fide* cell surface membrane proteins with one or more transmembrane domains or with a GPI anchor (Figure 1 and Table S1). Because our proteomic analysis was performed using pooled cells from different donors, 32 different alleles of the major histocompatibility complex (MHC) class I (HLA-A, HLA-B, and HLA-C) and MHC class 2 proteins were also identified. In addition, we identified 18 secreted and 14 intracellular proteins, which are potentially associated with ecto or cytoplasmic domains of these cell surface membrane proteins. Thus, our analysis identifies with high confidence ≈200 integral- and soluble proteins potentially associated with the plasma membrane that may regulate hMSC fate.

### Characterization of hMSC cell surface proteins

Classification of the ≈200 plasma membrane-associated proteins revealed several classes (Figure 1 and Table S1). Among these proteins, a large set comprised 33 proteins involved in cell adhesion like integrin chains α 1, 2, 3, 5, 7, 8, 11, V, β 1, 3, 5, cadherins 2, 11, 13, and some adhesion molecules of the immunoglobulin superfamily (e.g. CD54, CD106, and CD316). Our analysis also revealed another large set of 26 proteins comprising receptors involved in cell signaling such as PDGF-receptor β, EGF-receptor, TGF β–receptor, TNF-receptor 6, ephrin receptors, etc. In addition, we identified a panel of 21 transporters (e.g. solute carriers (1A5, 3A2, 7A5, 44A1, and 44A2) and ATPases (1A1, 1B1, 1B3, 2B1, 2B4, and 11c)), groups of 13 ubiquitously expressed receptors (e.g. Transferrin receptor, Anthrax toxin receptors 1 and 2, and Atrial natriuretic peptide receptors), 11 enzymes like the metalloproteases 10 and 14, or groups of tetraspanin and GPI-anchored proteins like the Thy-1 membrane protein. Secreted proteins most likely bound to the cell surface of hMSCs could also be identified, in particular several types of collagen, several lectins (galectins), or protease inhibitors (serpins). Finally, our data revealed intracellular proteins potentially associated with plasma membrane proteins via LIM domains or proteins known to interact with cell adhesion molecules like catenins binding to cadherins.

In our analysis we detected 41 predefined CD markers (Table 1 and Table S2). Thirty-one of these were already known as hMSC-related antigens. The presence of 5 CD markers (CD98, CD99, CD155, CD304, and CD325) at the hMSC surface was not firmly established. Therefore, our analysis confirms the expression of these CD markers. Interestingly, we also identified 5 CD markers (CD97, CD112, CD239, CD276, and CD316) that have never been reported to be expressed on the surface of hMSCs before.

**Table 1:** Expression of CD markers on human mesenchymal stromal cells.

Marker^b^^,^ c	MS			Flow cytometry	Literature^a^
	Protein expressed	Peptide numbers	Percentage coverage		Protein expressed
CD9: Tetraspanin–29	+	10	31.14	+	+
CD13^b^: Aminopeptidase N	+	74	29.99	nd	+
CD14: LPS receptor	−	−	−	−	−
CD29: Integrin β-1	+	145	40.48	+	+
CD34: Hematopoietic progenitor cell antigen	−	−	−	−	−
CD44: Phagocyte glycoprotein 1	+	139	13.61	+	+
CD45: Leukocyte common antigen	−	−	−	−	−
CD46: Membrane cofactor protein	+	6	8.67	nd	+
CD47^b^: Leukocyte surface antigen	+	4	5.88	nd	+
CD49a: Integrin α-1	+	39	16.96	nd	+
CD49b: Integrin α-2	+	3	2.96	nd	+
CD49c: Integrin α-3	+	18	10.23	nd	+
CD49e: Integrin α-5	+	31	13.25	nd	+
CD51: Integrin α-V	+	45	29.01	nd	+
CD54^b^: Intercellular adhesion molecule 1 (ICAM1)	+	5	8.27	+	+/−
CD56: Neural cell adhesion molecule (NCAM)	−	−	−	+	+/−
CD59: Protectin	+	12	25.78	nd	+
CD61^b^: Integrin β-3	+	4	7.74	(+)	+/−
CD63: LAMP-3	+	9	7.56	+	+
CD71: Transferrin receptor	+	21	25.53	+	+
CD73^b^: Ecto-5′-nucleotidase	+	24	29.44	+	+
CD81: Tetraspanin-28	+	47	33.05	nd	+
CD90: Thy-1	+	35	24.84	+	+
CD95^b^: Fas antigen	+	17	27.76	nd	+
CD97^b^: Leukocyte antigen CD97	+	10	9.58	+	nd
CD98: Solute carrier family 3 (SLC3A2)	+	98	52.55	+	+
CD99^b^: E2 antigen	+	21	15.14	+	+
CD105^b^: Endoglin	+	52	34.65	+	+
CD106^b^: Vascular cell adhesion molecule-1 (VCAM1)	+	29	16.78	+/−	+/−
CD109^b^: Platelet–specific Gov antigen	+	28	13.91	nd	
CD112: Polio virus receptor related 2 protein	+	14	14.13	(+)	nd
CD133: Prominin-1	−	−	−	−	−
CD140b^b^: Platelet derived growth factor	+	94	29.57	nd	+
CD146: Melanoma cell adhesion molecule (MCAM)	+	68	47.21	+	+
CD147: Basigin	+	56	25.71	nd	+
CD151^b^: Tetraspanin-24	+	49	20.95	nd	+
CD155^b^: Polio virus receptor	+	17	18.71	+	+
CD166: Activated leukocyte cell adhesion molecule (ALCAM)	+	92	48.54	+	+
CD172a^b^: Signal regulatory protein alpha	+	19	21.27	nd	+
CD239: Basal cell adhesion molecule (BCAM)	+	10	16.4	nd	nd
CD248: Endosialin	+	19	9.78	nd	+
CD276: B7 homolog 3	+	20	26.4	+	nd
CD304^b^: Neuropilin-1	+	7	4.98	+	+
CD316: Immunoglobulin superfamily, member 8	+	4	10.28	nd	nd
CD325: Cadherin 2	+	12	15.67	nd	nd (mRNA positive)

+ and − refer to the presence or absence of a given marker, respectively.

+/− refers to heterogenous expression within an hMSC population.

nd; not determined.

a References for the published results are presented in Table S2.

b CD markers unique for hMSCs compared with those for hESCs published by Dormeyer et al. [34].

c CD markers unique for hESCs: CD30, CD40, CD49f, CD74, CD133, CD200, CD266, CD271 and CD320 [34].

**Figure 2:**
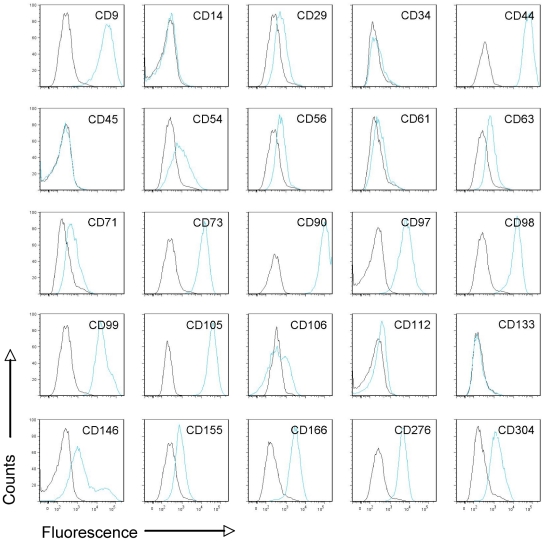
Flow cytometric analysis of hMSC surface CD markers. Cells were harvested and labeled with fluorescence-conjugated antibodies recognizing cell surface markers. Black: isotype control; blue: Ab against surface markers. At least three independent experiments were performed. Shown are representative flow cytometry histograms.

The MS-based proteomic identifications were confirmed by flow cytometry using a panel of specific antibodies recognizing some of the CD markers identified by MS. Our analysis demonstrated that the expanded, plastic-adherent hMSCs were positive for a number of surface markers (Figure 2). During all cell passages, the hMSCs were positive for the hMSC-associated CD markers CD29, CD44, CD73, CD90, CD105, CD146, and CD166, and negative for the hematopoietic markers CD14, CD34, and CD45 as well as CD133 as previously reported [33]. In addition, the hMSCs were positive for a panel of other surface markers like CD54, CD56, CD61, CD63, CD71, CD97, CD98, CD99, CD106, CD112, CD155, CD276 and CD304 in agreement with the MS data (except for CD56) (Figure 2, Table 1, and Table S2). Furthermore, CD325 expression was confirmed by western blot analysis using total cell extracts from hMSCs (data not shown).

### Cell surface proteomes of adult mesenchymal stromal cells and embryonic stem cells

**Figure 3:**
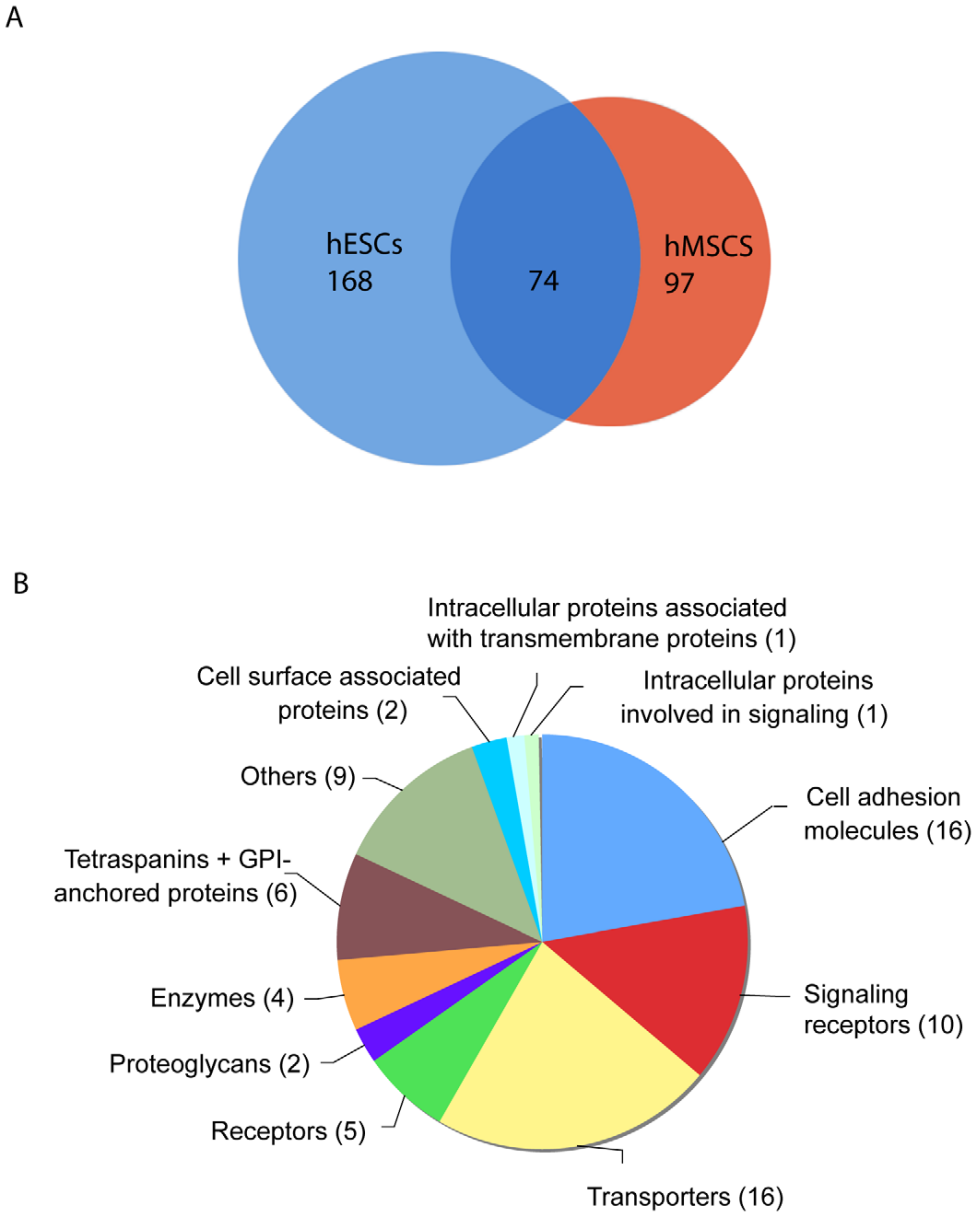
Comparison of hMSC and hESC cell surface protein profiles. A. Comparison of the membrane protein profiles of hMSCs (our study) and hESCs according to [34]. The diagram shows unique and common membrane proteins of hESCs (left) and hMSCs (right). B. Distribution and functional clustering of common membrane proteins between hESCs and hMSCs.

Our comprehensive analysis of the cell surface proteome of adult hMSCs allowed us to already establish with high confidence similarities and differences/changes in cell surface proteomes of different cell types. Of particular interest is the proteomic surface profile of hESCs, which comprises 242 membrane proteins identified thus far [34]. This comparison showed that surprisingly hMSCs and hESCs have only 74 proteins in common, whereas 97 and 168 proteins are unique for hMSCs and hESCs, respectively (Figure 3A and Table S3). Among these 74 common proteins, adhesion molecules, signaling receptors, and transporters are the most representative when compared to the total number of surface membrane proteins identified in hMSCs and hESCs (Figures 1 and 3B). Only 16 adhesion molecules (incl. 8 integrins (α 1, 2, 3, 5, 7, V, and β 1 and 5 chains)) among the 33 cell adhesion molecules identified on hMSCs were found on hESCs, thus suggesting that the other unique cell adhesion molecules could be considered as important molecules for specifying the proper niches of the corresponding stem cells. The same remark could be made for the proteoglycans detected at the cell surface of hMSCs and hESCs. Only 10 out of the 26 signaling receptors detected on hMSCs were found at the surface of hESCs such as EGF receptor, Ephrin type A receptor 2, or Ephrin type B receptor 4, thereby suggesting that the other signaling receptors are important for maintaining the stemness or determining the fate of hMSCs or hESCs. Finally, it is interesting to note that hMSCs and hESCs also express different CD markers (Table 1). Among the 41 hMSC CD markers, 16 of them (CD13, 47, 54, 61, etc.) were only detected on hMSCs and not on hESCs.

### Modifications of the hMSC cell surface proteome during adipogenesis and osteogenesis

**Figure 4:**
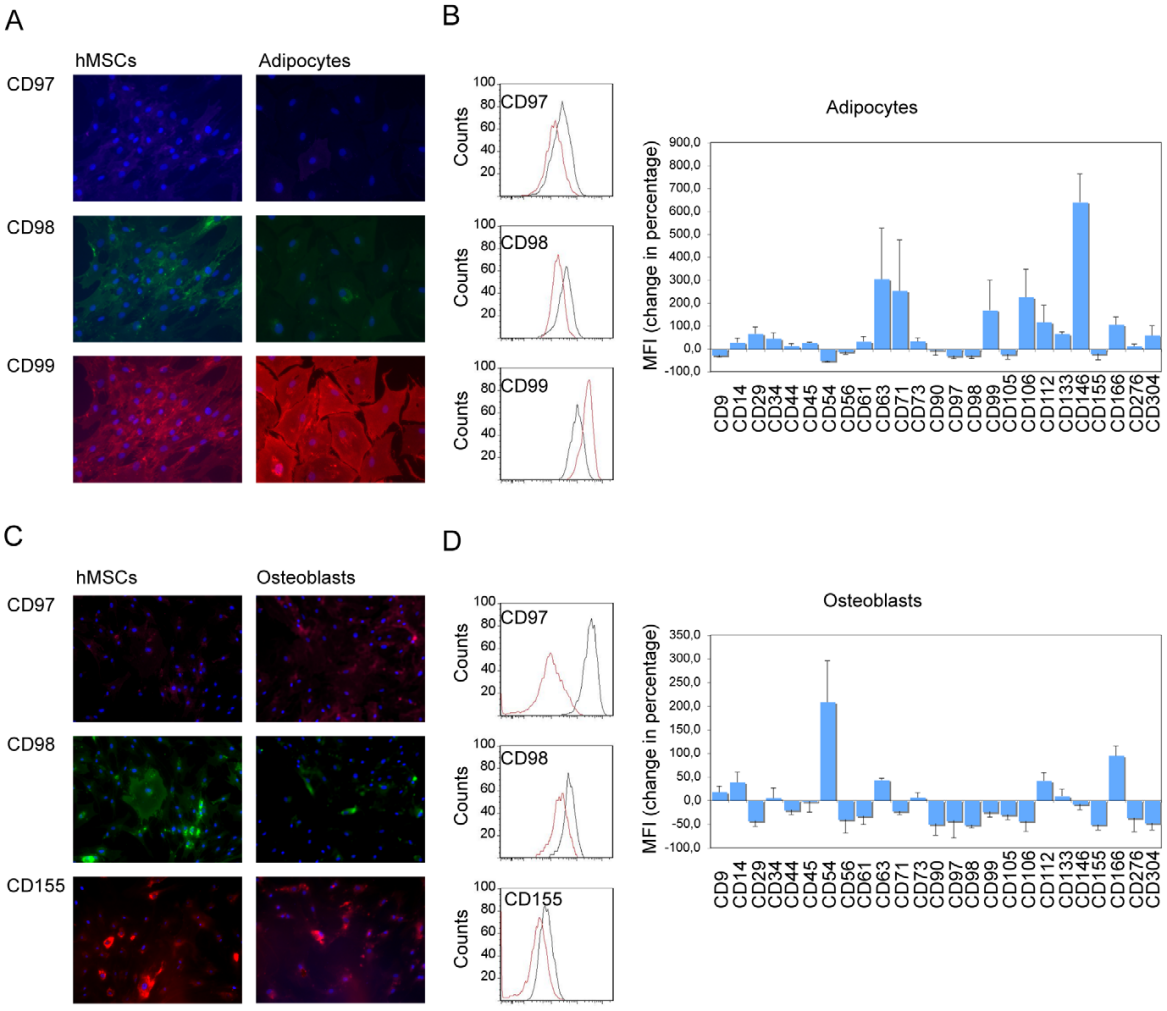
Differentiation of hMSCs towards the adipogenic or osteogenic lineage. **A+B**. Immunocytochemistry after 1 week of differentiation. Nuclei were visualized with DAPI. **C+D**. Flow cytometry after 2 weeks. The change in the mean fluorescence intensity (MFI) before and after differentiation was calculated taking hMSCs as a reference. At least three independent experiments were performed. Black: hMSCs; red: differentiated cells. Shown are representative stainings.

The hMSCs were differentiated toward the osteogenic or the adipocytic lineages using classical differentiation cocktails. After differentiation, modifications of the cell surface proteome, more precisely the CD markers, were monitored by flow cytometry and immunocytochemistry. These analyses showed that the expression of a panel of markers was changed (Figure 4). CD90, CD97, CD98, CD105, and CD155 were downregulated in both adipocytes and osteoblasts, whereas CD63, CD73. CD112, and CD166 were upregulated in both cell types after 2 weeks of differentiation. We noticed that CD97 expression increased during the first week of differentiation towards osteoblasts but decreased drastically when osteoblasts were mature. The remaining markers, CD9, CD29, CD44, CD54, CD56, CD61, CD71, CD99, CD106, CD146, CD276, and CD304 had different expression patterns during adipogenesis and osteogenesis, respectively, whereas the expression of the negative markers, CD14, CD34, CD45, and CD133, did not change considerably as they were already not detected on hMSCs (Figure 2).

## Discussion

This study identifying ≈200 *bona fide* cell surface proteins represents to our knowledge the most comprehensive cell surface proteome of plastic-adherent hMSCs available to date. Our study also highlighted with high confidence and good sequence coverage 5 new CD markers among the 41 identified, and how they change in expression after differentiation into adipocytes or osteoblasts. Thus, our data can be used for further isolation of hMSCs from bone marrow or other tissues and for monitoring changes in the cell surface proteome during the differentiation of hMSCs towards different cell lineages.

A large majority of the proteins identified in our analysis have never been reported to be present at the surface of hMSCs. This is most likely due to the limits of the methods used previously to isolate hMSCs, to purify membrane proteins or to identify cell surface proteins by MS. For example, several studies have used cell fractionation to purify microsomal membranes, which include not only plasma membranes but also many other intracellular membrane bounded compartments. For instance, Foster et al. who purified membranes on density gradients found only ≈75 *bona fide* cell surface proteins among the 463 proteins identified [27]. Similar analysis with total membranes led to the identification of ≈100 *bona fide* cell surface proteins among the 707 proteins identified [28]. Other proteomic analyses have been based on classical 2D-gel fractionation of total proteins [30], [31], [32], a method that is not optimal for the fractionation of high molecular weight membrane proteins, which do not focus well. One study included the labeling of intact cells with impermeable fluorescent dyes prior to 2D-gel analysis to identify cell surface proteins. However, this method had a much lower sensitivity than the non-gel approach performed in the same study [28]. In order to reduce the complexity of the proteome, we have chosen to use biotinylation of surface proteins followed by streptavidin affinity enrichment, as this method has previously been used to identify plasma membrane proteins and can be adapted for proteomic approaches [35], [36], [37]. For example, Gu et al. have identified plasma membrane proteins from murine ESCs using biotinylation [35]. In addition, we have used cleavable reagents allowing the release of biotinylated proteins while keeping many proteins non-specifically adsorbed onto the support. In addition, we have chosen well-defined culture conditions maintaining as much as possible the multipotency properties of plastic-adherent hMSCs [33]. Thus, our proteomic analysis identifying 201 *bona fide* plasma membrane proteins is therefore at least two times more comprehensive than previous studies, in particular when cell adhesion molecules, signaling receptors, and CD markers are considered.

Our proteomic analysis of cell surface proteins to a large extent confirmed by flow cytometry, identifies with high confidence a panel of 5 new markers of plastic adherent hMSCs that could be used for their prospective isolation from tissues. Among the identified proteins are several integrin chains (e.g. α 7, 8, and 11), and CD antigens like CD97, CD98, CD99, CD112, CD155, CD239, CD276, CD304, CD316, and CD325. Some of these markers were identified earlier in a few reports but their significance was still questioned [27], [28], [38], and the expression of CD97, CD112, CD239 CD276, and CD316 on hMSCs was not previously reported. Thus, our study resolves these ambiguities. These proteins may have an important role in hMSC biology. For example, the solute carrier, CD98, has a possible role in amino acid transport necessary for cell growth, and several proteins may be involved in cell adhesion such as CD97, CD99, CD112 (poliovirus receptor-related protein 2), CD155 (poliovirus receptor), and CD325 (cadherin-2) [39], [40]. Some others may be involved either in cell proliferation (e.g. CD276) or cell differentiation like the receptor CD304 (neuropilin-1) [28], [41]. Finally, CD316 interacts with the tetraspanins CD9 and CD81, which are also known hMSC markers [42], [43], [44]. However, our analysis failed to identify few known markers of hMSCs like CD56 or CD133 although our flow cytometry data indicated that the hMSCs were partially positive at least for CD56. It is known that both CD56 and CD133 are expressed in freshly isolated hMSCs and that their expression is rapidly down-regulated when hMSCs are maintained in culture [21], [42].

Among the different classes of cell surface proteins, the two major classes consisting of cell adhesion molecules and signaling receptors are more than likely playing a major role in hMSC biology, in particular for maintaining their stemness feature or triggering their differentiation towards different lineages. According to our analysis, hMSCs express on their surface 7 α and 3 β integrin chains, thereby providing a wide spectrum of possible integrin receptors, 3 cadherins (together with α, β and δ catenins for signaling) and other types of CD markers associated with cell-to-cell adhesion such as CD54, CD106, and CD316 of the immunoglobulin superfamily. Some of these cell adhesion molecules such as integrins α 1, 2, 3, 5, 7, and V, and β 1, 5 as well as CD44 and CD166 were also detected at the surface of hESCs whereas some others such as integrins α 8 and 11, and β 3, cadherins 2, 11, 13, CD97 or CD99 were found unique to hMSCs. A similar observation could be made for proteoglycans and secreted proteins such as collagens, galectins or fibronectin, which specify the nature of extracellular matrixes. This may indicate that a combinatorial use of adhesion molecules may be required for maintaining stem cells in a given niche. However, this possible interpretation waits for a more comprehensive understanding of the cell surface proteome of hESCs, for which our analysis of hMSCs may provide a new basis.

Roughly, 25 signaling molecules are present at the cell surface of hMSCs such as EGF receptor, ephrin type-B receptor 4, and ephrin type-A receptor 2 also detected on hESCs. TGF-β receptor type-1, PDGF receptor β, tyrosine-protein phosphatase non-receptor type substrate 1, and tumor necrosis factor receptor superfamily member 6 would be unique to hMSCs. Some of these receptors may be important for triggering the differentiation of hMSCs. EGF signaling is known to be important for triggering osteogenesis [45], and PDGF signaling is important for the chemotaxis of MSCs and osteoblasts during bone remodeling [46]. Ephrin type-B receptor 4 couples osteoblastogenesis and osteoclastogenesis, and its expression in MSCs from myeloma patients and in bone cells in myelomatous bones is lower than in healthy counterparts [47]. This receptor also regulates the embryonic stem cell differentiation [48]. Therefore, it is likely that the other signaling molecules detected in our proteomic screen of cell surface proteins of hMSCs play an important function in their biology. At the same time, some “stemness” genes might be downregulated during differentiation. Potential new stemness genes are CD97 and CD155. These genes were downregulated during both adipogenesis and osteogenesis in addition to their unique expression on hMSCs when compared to hESCs. CD97 with adhesive properties belongs to the epidermal growth factor-transmembrane 7 family and is known to play an important role in tumor differentiation, invasiveness, and metastasis by binding to its cellular ligand CD55 [49], [50], [51]. It has also been involved in leukocyte trafficking and function [52]. CD155, which serves as a ligand for CD226 and CD96 receptors is also overexpressed in tumor cell lines and primary tumors and promotes their invasion and migration [40], [53]. We also confirmed the downregulation of CD90, CD98, and CD105 as shown previously [28], [54]. Furthermore, we show the downregulation of a panel of markers (CD29, CD44, CD61, CD71, CD99, CD106, CD276, and CD304) during osteogenesis and downregulation of CD9 and CD54 during adipogenesis. It was also previously shown, that CD44, CD81, and CD166 are downregulated during chondrogenic differentiation [54], CD106 during osteogenic differentiation [55], and that the expression of some others is downregulated during several differentiation processes, for example integrin α 11 during osteogenic, adipogenic and chondrogenic differentiation, and CD325 during osteogenic and adipogenic differentiation [44]. We did not observe a complete loss of these markers, probably due to the fact, that the *in vitro* differentiation systems toward osteoblasts or adipocytes do not allow the differentiation of the total cell population.

Thus, our analysis of the cell surface proteome of hMSCs may provide key insights into their biology. It also provides a panel of membrane proteins, which can represent the basis for the definition of an hMSC signature that can be used for the identification and the isolation of hMSCs for regenerative medicine.

## Materials and Methods

### Isolation and culture of plastic–adherent hMSCs

Bone marrow aspirates were collected from healthy donors after verbal and written consent. The study was approved by the local ethics committee (Ethikkommission an der Technischen Universität Dresden, Ethic board no. EK263122004). hMSCs were isolated and cultured as previously described [33]. Briefly, 5–7 ml of bone marrow aspirate was diluted 1∶5 in phosphate-buffered saline (PBS) containing 0.5% human serum albumin (HSA; Braun, Melsungen, Germany). A 20-ml aliquot was layered over a Percoll solution (*d* = 1.073 g/ml; Biochrom, Berlin, Germany) and centrifuged at 900 *g* for 30 min at room temperature. Mononuclear cells at the interface were recovered, pressed through a 100 µm Nylon cell strainer (BD Biosciences, Heidelberg, Germany) and washed twice in PBS–HSA solution. All cells were seeded into 75-cm^2^ flasks containing MSC medium, consisting of Dulbecco's modified Eagle medium (DMEM)-low glucose (Invitrogen, Darmstadt, Germany) supplemented with 10 mM L-glutamine and 10% fetal calf serum (FCS) (Biochrom). hMSC cultures were grown at 37°C under a humidified 5% CO_2_ atmosphere. Nonadherent cells were removed after 24 h by washing with PBS–HSA solution. The medium was changed every 4 days and after 2 weeks the cultures were 90% confluent. hMSCs were recovered using trypsin (Invitrogen) and replated at a density of 5–6×10^3^ cells per cm^2^ of surface area as passage 1 cells. Subsequently cells were kept in culture for up to 8 passages and tested routinely for the presence of MSC-associated surface molecules like CD29, CD44, CD73, CD90, and CD105, and the absence of the hematopoietic markers CD34 and CD45. Under such conditions, hMSCs maintain their multilineage capacity to differentiate into osteoblasts, chondrocytes, and adipocytes [33].

For the differentiation assays, cells were seeded into 24-well plates at a density of 1×10^4^ cells per well. Next day the initial growth medium was replaced with medium for either adipogenic or osteogenic differentiation. The adipogenic medium consisted of DMEM-low glucose supplemented with 10 mM L-glutamine, 10% FCS, 500 µM isobutyl-methylxanthine, 1 µM dexamethasone, 10 µM insulin, and 200 µM indomethacin whereas the osteogenic medium was supplemented with 10 mM L-glutamine, 10% FCS, 0.1 µM dexamethasone, 200 µM ascorbic acid, and 10 mM β-glycerophosphate (all supplements were from Sigma-Aldrich, Munich, Germany). The level of adipogenic differentiation was tested by Oil red O staining of intracellular lipid droplets, and osteogenic differentiation was tested by Alizarin red S staining as described previously [56] (results not shown).

### Biotinylation of cell surface proteins

hMSCs (∼10^7^ cells from different donors) were rinsed three times with PBS supplemented with 1 mM CaCl_2_ and 0.5 mM MgCl_2_ (Ca/Mg-PBS), and incubated with 0.5 mg/ml EZ-Link Sulfo-NHS-SS-biotin (Thermo Fisher Scientific, Bonn, Germany) for 1 h at 4°C on a rocking platform. After washing three times with Ca/Mg-PBS, the cells were incubated with 20 mM glycine in Ca/Mg-PBS for 10 min to quench the residual biotin followed by three washes with Ca/Mg-PBS. The cells were harvested, centrifuged for 5 min at 500 *g*, and then lysed in 250 µl ice-cold PBS containing 0.1% SDS, 1% Triton X-100, and Protease Inhibitor Mix (Serva Electrophoresis, Heidelberg, Germany). This extract was diluted four-fold with Ca/Mg-PBS and incubated with 20 ml streptavidine-agarose beads for 2 h at 4°C while rotating. The beads were washed five times with Ca/Mg-PBS containing 0.1% Triton X-100 and five times with five-fold diluted Ca/Mg-PBS containing 0.1% Triton X-100. Then the beads were incubated with elution buffer containing 50 mM 2-mercaptoethanesulfonate in Ca/Mg-PBS supplemented with 0.1% Triton X-100 for 2×30 min at RT. The released material was transferred into an Ultrafree-MC spin filter (0.45 µm) (Millipore, Schwalbach, Germany) and centrifuged in a table-top centrifuge. The proteins in the flow through were precipitated with 10% trichloroacetic acid, and the pellet obtained after centrifugation was dissolved in Laemmli buffer.

### Sample preparation, mass spectrometric analysis and protein identification

The biotinylated proteins were fractionated onto a Tris-glycine PAGE gel and stained with Coomassie G-250. The lanes were cut into 24 slices. The embedded proteins were reduced with dithiothreitol, alkylated with iodoacetamide, digested overnight with trypsin in a 1∶50 ratio and subjected to LC-MS/MS analysis. Peptides were separated on an EASY-nLC HPLC system (Proxeon, Odense, Denmark) equipped with a fused silica microcapillary C18 column (Proxeon, length 10 cm; inner diameter 75 µm; particle size 3 µm, 100 Å pore size. The gradient used was: A, 0.1% formic acid; B, acetonitrile, 0.1% formic acid with a final concentration of 80% B. Mass spectrometry analysis was made on a LTQ Orbitrap XL mass spectrometer (Thermo Fisher Scientific). The MS data were analyzed using the Proteome Discoverer 1.0 software (Thermo Fisher Scientific). Mascot (version 2.2.2) and the SwissProt database (SwissProt_56.9.fasta) were used for interpretation of spectra applying the following settings: the taxonomy was set to human and trypsin as the enzyme allowing up to two missed cleavages. Precursor mass tolerance was set to 10 ppm, fragment mass tolerance to 0.5 Da. As a static modification carbamidomethylation (of Cysteine) was chosen and as dynamic modifications deamidation (of Asparagine and Glutamine) and oxidation (of Methionine). Protein hits were filtered for a minimum of identified peptides of two with a minimum score of 40, possessing either transmembrane domains and known plasma membrane localization or a signaling sequence and known lipid modification.

### Flow cytometry

Flow cytometric analysis was performed on hMSCs harvested by cell scraping or trypsin (Invitrogen) treatment. After washing cells in ice-cold PBS containing 2% FCS, the appropriate antibodies were added, and the stainings were performed in 50 µl cell suspension for 30 min at 4°C. The following antibodies were used: CD9-PE (clone HI9a), CD90-Alexa700 (clone 5E10) (both from BioLegend, Uithoorn, The Netherlands), CD14-APC (clone 61D3), CD97-APC (clone VIM3b), CD99-PE (clone 3B2/TA8), CD112-APC (clone R2.525) (all from eBioscience, San Diego, CA, USA), CD29-PE (clone HUTS-21), CD34-FITC (clone 581), CD44-PE (clone G44-26), CD45-APC (clone HI30), CD54-Alexa488 (clone HA58), CD56-PE (clone B159), CD61-FITC (clone VIPL2), CD71-FITC (clone M-A712), CD73-PE (clone AD2), CD98-FITC (clone UM7F8), CD106-FITC (clone 51-10C9), CD166-PE (clone 3A6), (all from BD Biosciences), CD63-FITC (clone MEM-259) (from Acris Antibodies, Herford, Germany), CD146-APC (clone 541-10B2), CD133-PE (clone 293C3) (both from Miltenyi Biotec, Bergisch Gladbach, Germany), CD155-PE (clone 300907), CD276 (B7-H3)-Fluorescein (clone 185504), and CD304 (neuropilin-1)-Fluorescein (clone 446921) (all from R&D Systems, Wiesbaden-Nordenstadt, Germany). CD105-APC (clone SN6) was purchased from Invitrogen. Mouse IgG1-Alexa Fluor 488 and mouse IgG1-Alexa Fluor 700 isotype controls were from eBioscience. Otherwise all isotype controls were from BD Biosciences. Except for the stainings with antibodies against CD61, CD71, and CD304, where the cells were scraped off the culture plate, all data was acquired after trypsinization of the cells. After washing with PBS containing 2% FCS, cells were acquired on an LSRII flow cytometer (BD Biosciences). Instrument settings and gating strategies were established using isotype controls. Data was analyzed using the FlowJo software (Treestar, Ashland, OR, USA).

### Immunocytochemistry

hMSCs, differentiated adipocytes and osteoblasts grown on coverslips were cell-surface immunolabeled. Briefly, cells were washed twice with PBS followed by ice-cold Ca/Mg-PBS, and then incubated with fluorescence-conjugated antibodies diluted in Ca/Mg-PBS for 1 h at 4°C. Labeled cells were washed twice with PBS and fixed with 4% paraformaldehyde for 15 min at RT. After quenching with 50 mM NH_4_Cl for 10 min, fixed cells were washed with PBS and mounted in Mowiol (Merck, Darmstadt, Germany) containing 4,6-diamidino-2-phenylindole (DAPI) for counterstaining the nuclei. The resulting stainings were examined using a Zeiss Axiovert 200 M fluorescence microscope.

## Supporting Information

Table S1 List of hMSC cell surface proteins identified in this study.
(XLS)

Table S2 Reference list for Table 1.
(DOC)

Table S3 List of hESC and hMSC cell surface proteins compared in this study.
(XLS)
