# Supplementary material for: The Cell Surface Proteome of Human Mesenchymal Stromal Cells
Source: PLoS One. 2011 May 26;6(5):e20399. doi: 10.1371/journal.pone.0020399 (PMC3102717; doi:10.1371/journal.pone.0020399)
Supplement: Table S2 — Reference list for Table 1. (DOC) [file pone.0020399.s002.doc]

Table S2.

References to Table 1.

| Marker | References, expression in hMSCs |
| --- | --- |
| CD9 | [1] |
| CD13 | [2] |
| CD14 | [3] |
| CD29 | [2] |
| CD34 | [4] |
| CD44 | [2,5] |
| CD45 | [4] |
| CD46 | [6] |
| CD47 | [7] |
| CD49a | [8] |
| CD49b | [8] |
| CD49c | [2,8] |
| CD49e | [2,8] |
| CD51 | [2,8] |
| CD54 | [9] |
| CD56 | [1] |
| CD59 | [2,10] |
| CD61 | [8] |
| CD63 | [2,11] |
| CD71 | [2] |
| CD73 | [2] |
| CD81 | [2,5,12] |
| CD90 | [2,5] |
| CD95 | [13] |
| CD97 | [14] |
| CD98 | [2,15] |
| CD99 | [15] |
| CD105 | [2,5] |
| CD106 | [5,9,16] |
| CD109 | [2] |
| CD112 | [17] |
| CD133 | [18] |
| CD140b | [2,19] |
| CD146 | [2,20] |
| CD147 | [2,21] |
| CD151 | [2,12] |
| CD155 | [2,22] |
| CD166 | [2,9] |
| CD172a | [19] |
| CD239 | - |
| CD248 | [23] |
| CD276 | [24] |
| CD304 | [2] |
| CD316 | [25] |
| CD325 | [5] |

References

1. Battula VL, Treml S, Bareiss PM, Gieseke F, Roelofs H, et al. (2009) Isolation of functionally distinct mesenchymal stem cell subsets using antibodies against CD56, CD271, and mesenchymal stem cell antigen-1. Haematologica 94: 173-184.

2. Jeong JA, Ko KM, Park HS, Lee J, Jang C, et al. (2007) Membrane proteomic analysis of human mesenchymal stromal cells during adipogenesis. Proteomics 7: 4181-4191.

3. Dominici M, Le Blanc K, Mueller I, Slaper-Cortenbach I, Marini F, et al. (2006) Minimal criteria for defining multipotent mesenchymal stromal cells. The International Society for Cellular Therapy position statement. Cytotherapy 8: 315-317.

4. Tarnok A, Ulrich H, Bocsi J (2010) Phenotypes of stem cells from diverse origin. Cytometry A 77: 6-10.

5. Song L, Webb NE, Song Y, Tuan RS (2006) Identification and functional analysis of candidate genes regulating mesenchymal stem cell self-renewal and multipotency. Stem Cells 24: 1707-1718.

6. Pessina A, Bonomi A, Cocce V, Bernardo ME, Cometa AM, et al. (2009) Assessment of human herpesvirus-6 infection in mesenchymal stromal cells ex vivo expanded for clinical use. Transpl Infect Dis 11: 491-496.

7. Goessler UR, Bugert P, Bieback K, Stern-Straeter J, Bran G, et al. (2008) Integrin expression in stem cells from bone marrow and adipose tissue during chondrogenic differentiation. Int J Mol Med 21: 271-279.

8. Semon JA, Nagy LH, Llamas CB, Tucker HA, Lee RH, et al. (2010) Integrin expression and integrin-mediated adhesion in vitro of human multipotent stromal cells (MSCs) to endothelial cells from various blood vessels. Cell Tissue Res.

9. Lee HJ, Choi BH, Min BH, Park SR (2009) Changes in surface markers of human mesenchymal stem cells during the chondrogenic differentiation and dedifferentiation processes in vitro. Arthritis Rheum 60: 2325-2332.

10. Han K, Lee JE, Kwon SJ, Park SY, Shim SH, et al. (2008) Human amnion-derived mesenchymal stem cells are a potential source for uterine stem cell therapy. Cell Prolif 41: 709-725.

11. Zannettino AC, Harrison K, Joyner CJ, Triffitt JT, Simmons PJ (2003) Molecular cloning of the cell surface antigen identified by the osteoprogenitor-specific monoclonal antibody, HOP-26. J Cell Biochem 89: 56-66.

12. Diaz-Romero J, Nesic D, Grogan SP, Heini P, Mainil-Varlet P (2008) Immunophenotypic changes of human articular chondrocytes during monolayer culture reflect bona fide dedifferentiation rather than amplification of progenitor cells. J Cell Physiol 214: 75-83.

13. Dimitriou H, Perdikogianni C, Martimianaki G, Choumerianou DM, Pelagiadis J, et al. (2009) Are mesenchymal stromal cells from children resistant to apoptosis? Cell Prolif 42: 276-283.

14. van Pel M, Hagoort H, Hamann J, Fibbe WE (2008) CD97 is differentially expressed on murine hematopoietic stem-and progenitor-cells. Haematologica 93: 1137-1144.

15. Foster LJ, Zeemann PA, Li C, Mann M, Jensen ON, et al. (2005) Differential expression profiling of membrane proteins by quantitative proteomics in a human mesenchymal stem cell line undergoing osteoblast differentiation. Stem Cells 23: 1367-1377.

16. Liu F, Akiyama Y, Tai S, Maruyama K, Kawaguchi Y, et al. (2008) Changes in the expression of CD106, osteogenic genes, and transcription factors involved in the osteogenic differentiation of human bone marrow mesenchymal stem cells. J Bone Miner Metab 26: 312-320.

17. Deng HP, Zhuang R, Song CJ, Tian Y, Jia W, et al. (2007) [Preparation and characterization of monoclonal antibodies against human CD112 (Nectin2/PRR2)]. Xi Bao Yu Fen Zi Mian Yi Xue Za Zhi 23: 356-358.

18. Pozzobon M, Piccoli M, Ditadi A, Bollini S, Destro R, et al. (2009) Mesenchymal stromal cells can be derived from bone marrow CD133+ cells: implications for therapy. Stem Cells Dev 18: 497-510.

19. Vogel W, Grunebach F, Messam CA, Kanz L, Brugger W, et al. (2003) Heterogeneity among human bone marrow-derived mesenchymal stem cells and neural progenitor cells. Haematologica 88: 126-133.

20. Buhring HJ, Treml S, Cerabona F, de Zwart P, Kanz L, et al. (2009) Phenotypic characterization of distinct human bone marrow-derived MSC subsets. Ann N Y Acad Sci 1176: 124-134.

21. Mayer H (2004) Properties of human trabecular bone cells from elderly women: implications for cell-based bone engraftment. Cells Tissues Organs 177: 57-67.

22. Willmann JK, Paulmurugan R, Rodriguez-Porcel M, Stein W, Brinton TJ, et al. (2009) Imaging gene expression in human mesenchymal stem cells: from small to large animals. Radiology 252: 117-127.

23. Bagley RG, Weber W, Rouleau C, Yao M, Honma N, et al. (2009) Human mesenchymal stem cells from bone marrow express tumor endothelial and stromal markers. Int J Oncol 34: 619-627.

24. Zhang G, Hou J, Shi J, Yu G, Lu B, et al. (2008) Soluble CD276 (B7-H3) is released from monocytes, dendritic cells and activated T cells and is detectable in normal human serum. Immunology 123: 538-546.

25. Kettner S, Kalthoff F, Graf P, Priller E, Kricek F, et al. (2007) EWI-2/CD316 is an inducible receptor of HSPA8 on human dendritic cells. Mol Cell Biol 27: 7718-7726.
